# Uterine Rotation: A Cause of Intestinal Obstruction

**DOI:** 10.1155/2013/759250

**Published:** 2013-05-23

**Authors:** Ernesto González-Mesa, Isidoro Narbona, Isaac Cohen, Emilia Villegas, Celia Cuenca

**Affiliations:** ^1^Obstetrics and Gynecology Department, University Hospital Carlos Haya, Málaga, Spain; ^2^Málaga Research Group in Obstetrics and Gynecology, Biomedical Research Institute of Málaga (IBIMA), Spain; ^3^Servicio de Obstetricia y Ginecología, Hospital Materno Infantil, Arroyo de los Angeles Avenida s/n, 29006 Málaga, Spain

## Abstract

Intestinal obstruction is an uncommon surgical emergency during pregnancy that affects seriously the prognosis of gestation. The underlying cause can be identified in the majority of cases and usually consists of adhesions secondary to previous abdominal or pelvic surgery, followed in order of frequency by intestinal volvuli. In recent years there have been no reports in which the gravid uterus has been the cause of intestinal obstruction. We report the case of a woman in week 33 + 4 of pregnancy who developed extrinsic compression of the colon secondary to uterine rotation and pelvic impaction of the head of the fetus.

## 1. Background

Intestinal obstruction is an uncommon surgical emergency during pregnancy that affects the prognosis of gestation. Although maternal and fetal mortality associated with intestinal obstruction during pregnancy has decreased substantially in recent years, it is estimated that the maternal mortality rate is close to 6%, while 20–26% of the affected fetuses die in utero [1]. 

In the second half of pregnancy the clinical diagnosis is difficult to establish, because both abdominal distension and displacement of the abdominal organs as a result of uterine growth can mask the symptoms of obstruction. 

We report the case of a woman in week 33 + 4 of pregnancy who developed extrinsic compression of the colon secondary to uterine rotation and pelvic impaction of the head of the fetus. 

## 2. Clinical Case

An 18-year-old Caucasian female reported to the emergency service of our hospital in week 33 + 4 of her first pregnancy due to severe pain of sudden onset, located in the epigastric and left hypochondriac zone. The pain was described as continuous but with exacerbations. Previously the patient had been asymptomatic, with normal bowel habits, though upon questioning she explained that in the last two days the stools had been somewhat looser and scarcer than usual. 

Before this episode the patient had been healthy, with normal menstruation and no medical or surgical antecedents of interest. Pregnancy up until that point had been normal. Upon admission, she weighed 52 kg, measured 155 cm in height, and had gained 10 kg in the course of pregnancy. 

The vital functions were normal, with a temperature of 36°C and blood pressure 125/51 mmHg. Physical examination revealed a soft abdomen with pain in response to palpation in the epigastric and left hypochondriac zone but no signs of peritonism. The gynecological examination was normal, and the uterine cervix showed no modifications. The obstetric ultrasound findings were also normal, showing a fetus with longitudinal, cephalic presentation. The ultrasound measurements were consistent with dates, with normal placental insertion and amniotic fluid in normal quantities. The cardiotocographic recordings showed a reactive pattern with no contractions.

A peripheral venous catheter was inserted to administer analgesics, obtaining a very limited response. After the first hours of observation the pain proved more intense, irradiating to the subcostal region and left side of the chest. The blood tests showed a leukocyte count of 15,480 cells/mm^3^ (82.3% neutrophils), 10.9 g/dL hemoglobin and 269,000 platelets/mm^3^. The biochemical study was normal, with Na 143 mEq/L, K 4.2 mEq/L, and Cl 107 mEq/L. The transaminase, alkaline phosphatase, and amylase levels were within normal limits. Urine testing likewise showed no alterations.

**Figure 1:**
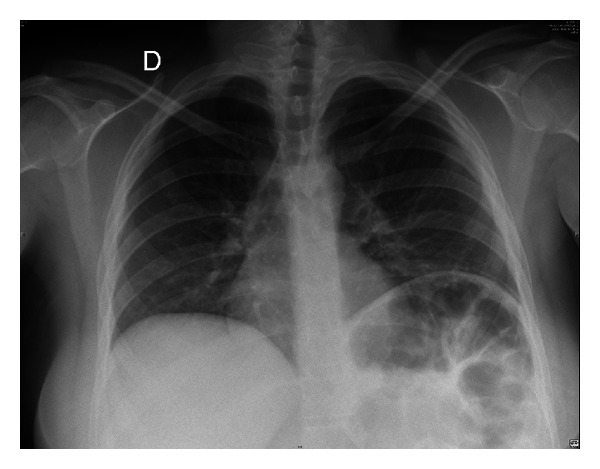
Chest X-ray showing left hemidiaphragmatic elevation due to the accumulation of gas in the splenic angle of the colon.

Abdominal ultrasound initially revealed no renal or liver alterations. The chest X-ray study showed left hemi-diaphragmatic elevation secondary to abundant abdominal gas in the splenic angle of the colon, with normal lung parenchyma and no alterations of the cardiomediastinal silhouette (Figure 1).

**Figure 2:**
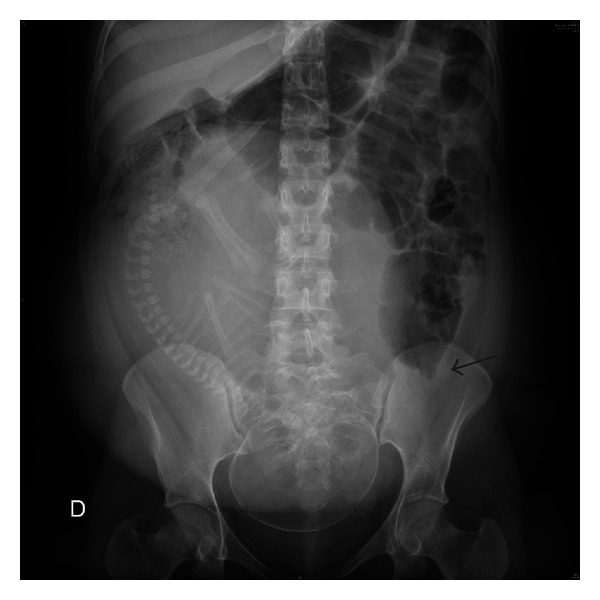
Abdominal X-ray showing dilatation of the small and large bowel loops. The arrow shows the stop at colon level. Displacement of the gravid uterus to the right is clearly seen.

A rectal tube accompanied by postural changes and spasmolytic agents did not facilitate evacuation of the gas. Given the gradual worsening of the pain, a plain X-ray study of the abdomen was carried out, revealing notorious displacement of the gravid uterus to the right, important dilatation of the jejunal loops, and a redundant descending colon containing abundant gas. Colon obstruction was noted at pelvic level, consistent with obstructive ileus secondary to impaction of the fetal head in the dextroflexed uterus (Figure 2).

Nasogastric intubation likewise failed to lessen the pain or facilitate intestinal evacuation. The general condition of the patient deteriorated as a result of intense pain. Accordingly, after 5 hours without improvement, and in view of the risk of bowel perforation as a result of the intense intestinal dilatation, we decided to perform an exploratory laparotomy, with possible fetal extraction. Betamethasone (one 12 mg dose) was administered to favor fetal pulmonary maturation. Surgery confirmed the presence of a dextroflexed uterus rotated about 90 degrees to the right, causing impaction of the head of the fetus over the sigmoid portion of the colon, compressing the lumen and producing the obstruction. There were no adherences or ischemic areas in the bowel loops suggesting other possible causes of obstruction. Cesarean section was performed, delivering a male weighing 1940 grams and with an Apgar score of 8/9.

**Figure 3:**
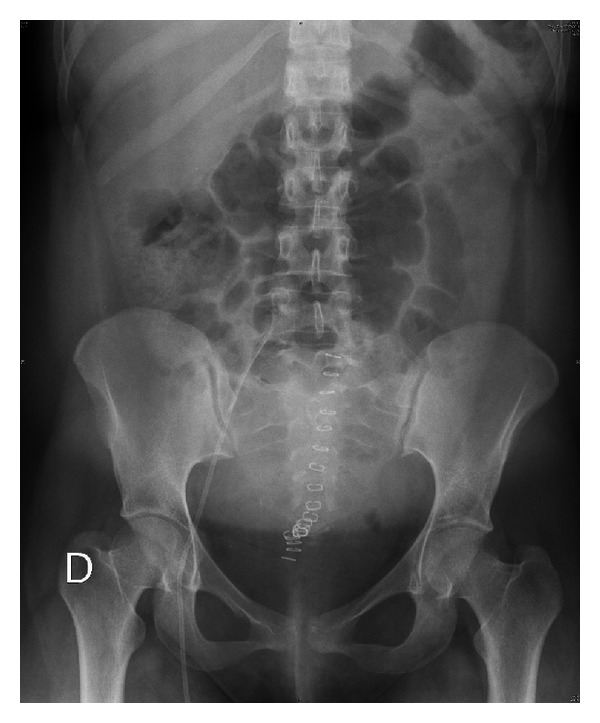
Abdominal X-ray on day three after cesarean section.

The puerperal course was favorable, and 72 hours after cesarean section the patient presented complete intestinal transit, allowing us to remove the nasogastric tube and resume oral feeding (Figure 3). The subsequent digestive study after puerperium did not reveal any intestinal pathology.

## 3. Discussion

Bowel obstruction is an infrequent complication of pregnancy. The incidence reported in the literature [2] varies between 1/1500 and 1/66,000. In most cases the underlying cause can be identified, the presence of adhesions secondary to previous abdominal or pelvic surgery being the most frequent etiology (58%), followed by volvuli (24%) and intussusception (5%). There are also a number of idiopathic obstructions in which the underlying cause cannot be established. Such presentations include transient and self-limiting intestinal dilatations and Ogilvie's syndrome, characterized by acute, nonobstructive dilatation of the colon. Ogilvie's syndrome is not a characteristic of pregnancy, though it has been described with some frequency in the puerperal period [3], particularly after cesarean section and in women at risk of preterm delivery treated with a combination of tocolytic drugs such as beta-adrenergic agents, nifedipine, and magnesium sulfate [4]. 

In our patient, rotation and flexion of the gravid uterus facilitated impaction of the fetal head against the intestinal lumen. Bowel obstruction as a result of normal pregnancy has been previously described in the first half of the last century [5]. It is also known that intestinal symptoms can develop in cases of uterine torsion [6]. But rotation has not been well documented before as a direct cause of bowel obstruction. Rotation of the uterus is common during pregnancy but rarely exceeds 45 degrees [7]. 

In recent years there have been reports of intestinal obstruction in pregnant women caused by adhesions [8–11], volvuli [12, 13] and hernias [14], and even cases secondary to retraction due to fibrin clots in the context of uterine rupture [15] though there have been no recent documented descriptions of bowel obstruction caused by the rotation of the gravid uterus.

From the clinical perspective, the symptoms of intestinal obstruction in pregnancy do not differ from those observed in nonpregnant women. The condition characteristically involves pain caused by dilatation of the intestinal lumen, accompanied by constipation, nausea, and vomiting. Initial treatment is conservative, with nasogastric aspiration and electrolyte replacement. When these measures prove ineffective and the condition worsens, surgery is indicated.

The lack of a diagnosis and adequate treatment causes loop dilatation to progress towards perforation and peritonitis secondary to release of the bowel contents into the peritoneal cavity. This is a serious condition with potentially fatal consequences for the patient and fetus. 

## 4. Conclusions

Rotation of the gravid uterus favors extrinsic intestinal compression and may produce complete intestinal obstruction. In the event of complete or progressive obstruction despite conservative management, fetal extraction through emergency cesarean section resolves the problem and avoids perforation secondary to dilatation of the bowel loops.
